# Experience with intraoperative neuromonitoring of the recurrent laryngeal nerve improves surgical skills and outcomes of non-monitored thyroidectomy

**DOI:** 10.1007/s00423-016-1449-5

**Published:** 2016-05-21

**Authors:** Beata Wojtczak, Krzysztof Sutkowski, Krzysztof Kaliszewski, Mateusz Głód, Marcin Barczyński

**Affiliations:** 1 Department and Clinic of General, Gastroenterological and Endocrine Surgery, Wroclaw Medical University, M.C. Sklodowskiej 66, 50-369 Wrocław, Poland; 2 Department of Endocrine Surgery, Third Chair of General Surgery, Jagiellonian University Medical College, Kraków, Poland

**Keywords:** Intraoperative neuromonitoring, Thyroid surgery, Recurrent laryngeal nerve, Surgical skill

## Abstract

**Purpose:**

Intraoperative neuromonitoring (IONM) can serve as a tool to increase skills in recurrent laryngeal nerve (RLN) identification and complete removal of thyroid tissue. The aim of this study was to validate this hypothesis.

**Methods:**

This prospective study involved 632 patients (1161 RLNs at risk) who underwent thyroid surgery in 2011–2014. Although IONM was not used until 2012, this prospective study started on 1 January 2011. The three participating surgeons knew about the study before that date and that the rate of RLN identification would be carefully measured in total and near-total surgery. Solely, visual identification of the RLN was used throughout 2011. IONM was introduced as a training tool in 2012–2014 for the first 3 months of each year. In the remaining months, thyroid operations were performed without IONM. Outcomes of non-monitored thyroid operations were compared before (01-12/2011) vs. after (04-12/2012–2014) 3 months of exposure to IONM yearly (01-03/2012–2014). The rate of RLN identification was assessed in total and near-total thyroidectomies and in totally resected lobes in Dunhill’s operation. The prevalence of RLN injury and the utilization of total thyroidectomy were evaluated.

**Results:**

In 2011, the rate of successful RLN visual identification in total and near-total thyroidectomies and in totally resected lobes in Dunhill’s operation was 45.71 %. After the introduction of IONM in 2012–2014, in the procedures performed without IONM, the rate was 86.66, 90.81, and 91.3 %. The prevalence of RLN injury in 2011 was 6.8 %, while in the years following the introduction of IONM, it was 3.61, 2.65, and 1.45 %. Utilization of total thyroidectomy increased from 47.9 % in 2011 to 100 % in 2014.

**Conclusions:**

Experience with IONM led to an increase in RLN identification (*p* < 0.0001), a decrease of RLN injury (*p* < 0.05), and an increase in the safe utilization of total thyroidectomy (*p* < 0.0001) in non-monitored thyroid operations. IONM is a valuable tool for surgical training.

## Introduction

Recurrent laryngeal nerve (RLN) paresis is a serious complication of thyroid surgery, which can significantly deteriorate the quality of life [1–4]. Intraoperative RLN identification during thyroid surgery reduces the risk of accidental injury and should be routinely performed during every operation [4–7].

Currently, identification of the RLN can be facilitated with intraoperative neuromonitoring (IONM), which is more and more widely accepted as a standardized method and utilized at centers for thyroid surgery [7–10]. Neuromonitoring is a tool that not only helps to visually identify the RLN but also predicts postoperative nerve function, which is a huge advantage over visualization alone; this technique could help prevent bilateral palsy [5]. Recently, there have been numerous publications assessing the value of IONM in thyroid surgery, comparing the effect of these procedures with and without the use of IONM [5, 11]. Considerably, fewer publications have evaluated the educational value of neuromonitoring as a tool in increasing surgeons’ insight into the operating field and skill at identifying the RLN [7, 29]. Moreover, it is worth considering whether the experience of working with IONM, even short term, can affect the quality of thyroid operations performed later—even those carried out without neuromonitoring.

The aim of this study was to validate the hypothesis that IONM can serve as a tool for increasing skills in RLN identification and safe, complete removal of thyroid tissue.

## Material and methods

A total of 632 consecutive thyroidectomy patients treated at the Department of General, Gastroenterological and Endocrine Surgery of Wroclaw Medical University in Wroclaw, Poland, between January 2011 and December 2014 were found to be eligible for this prospective study. Although IONM was not used until 2012, this prospective study started on 1 January 2011. The three participating surgeons knew about the study before that date and that the rate of RLN identification would be carefully measured in total and near-total surgery. Solely, visual identification of the RLN was used in 2011. IONM was used as a training tool for the first 3 months of 2012, 2013, and 2014. In the remaining months of each year, thyroid operations were performed without IONM (Table 1). The outcomes of non-monitored thyroid operations were compared in two time periods: before (01-12/2011) vs. after (04-12/2012–2014) 3 months of exposure to IONM yearly (01-03/2012–2014). The primary endpoint was RLN identification, while the secondary endpoints were the prevalence of RLN injury and the utilization of total thyroidectomy. The study was approved by the Bioethics Committee of Wroclaw Medical University.

**Table 1:** Demographic data, indications for surgery, and selected details of the procedures

	Visual RLN identification (2011, and 04-12/2012-2014)	RLN identification with IONM (01-03/2012–2014)	*p* value	Visual RLN identification 2011	Visual RLN identification (01-03/2012)	Visual RLN identification (01-03/2013)	Visual RLN identification (01-03/2014)	*p* value
No. of patients	396	236	NC	119	93	108	76	NC
RLNs at risk	727	434	NC	235	166	188	138	NC
Mean age ± SD, years	53.88 ± 14.43	53.99 ± 13.36	0.925^a^	55.36 ± 14.00	55.24 ± 14.09	51.58 ± 14.18	53.16 ± 15.65	0.012^b^
Sex ratio (F:M)	4.3:1	4.6:1	0.752^c^	3.8:1	5.6:1	5:1	3.2:1	0.436^d^
Nodular goiter, no. (%)	257 (64.9 %)	166 (70.34 %)	0.163^c^	83 (69.75 %)	60 (64.52 %)	66 (61.11 %)	48 (63.16 %)	0.320^d^
Thyroid cancer, no. (%)	27 (6.82 %)	35 (14.83 %)	0.001^c^	7 (5.88 %)	4 (4.30 %)	9 (8.34 %)	7 (9.21 %)	
Grave’s disease, no. (%)	11 (2.77 %)	9 (3.81 %)	0.488^c^	1 (0.84 %)	3 (3.23 %)	2 (1.85 %)	5 (6.58 %)	
Toxic nodular goiter, no. (%)	101 (25.51 %)	26 (11.02 %)	<0.001^c^	28 (23.53 %)	26 (27.95 %)	31 (28.7 %)	16 (21.05 %)	
Primary surgery, no. (%)	372 (93.94 %)	199 (84.32 %)	0.001^c^	108 (90.76 %)	86 (92.47 %)	104 (96.3 %)	74 (97.37 %)	0.165^d^
Secondary surgery, no. (%)	24 (6.06 %)	37 (15.68 %)		11 (9.24 %)	7 (7.53 %)	4 (3.7 %)	2 (2.63 %)	
Goiter’s volume, mean ± SD, ml	38.56 ± 29.13	41.25 ± 34.79	0.466^e^	43.87 ± 35.84	42.57 ± 35.24	38.4 ± 30.74	38.8 ± 26.87	0.171^b^
Retrosternal goiter, no. (%)	79 (19.94 %)	52 (22.03 %)	0.612^c^	23 (19.33 %)	18 (19.35 %)	22 (20.37 %)	16 (21.05 %)	0.989^d^
Compressio tracheae, no. (%)	177 (80.06 %)	112 (47.45 %)	0.510^c^	52 (43.7 %)	47 (50.54 %)	37 (34.26 %)	40 (52.63 %)	0.045^d^
Operating time, mean ± SD, min	108.5 (±30.47)	111.7 (±31.37)	0.596^c^	96.3 (±25.44)	121.3 (±20.6)	107.8 (±34.08)	93.82 (±28.56)	<0.001^f^

*p* value <0.05 was considered statistically significant

*IONM* intraoperative neuromonitoring, *RLN* recurrent laryngeal nerve, *NC* not calculated

^a^ *t* test

^b^ANOVA test

^c^Fisher’s exact test

^d^Chi-square test

^e^Mann- Whitney test

^f^Kruskall- Walis test

All the patients enrolled in the study were comprehensively diagnosed preoperatively and prepared for surgery by the Department or by the outpatient Endocrinology Clinic, and all of them were euthyroid. A greatly enlarged thyroid gland in the course of a goiter, compression symptoms and suspicion or diagnosis of a malignant thyroid tumor was the indications for surgical treatment of these patients. All the patients’ thyroid gland function was reassessed upon admission to the hospital, determining TSH and FT4 levels; routine chest and neck X-rays were done to assess displacement, narrowing of the trachea, and the presence of a retrosternal goiter. Other tests in the preoperative period were typical of the standard preparation of patients for any operating procedure. Before each operation, the patient underwent ENT examination of the vocal cords (indirect examination or videolaryngoscopy).

All the thyroid operations were performed by the same three surgeons (mean age 41 years old) with similar experience in thyroid surgery, performing about 60 thyroid operations a year. None of the surgeons participating in the study had much experience in RLN identification.

Before the initial use of the new technique, the surgical team was trained on a 2-day practical introductory course in neuromonitoring at the Department of Endocrine Surgery of Jagiellonian University Medical College in Krakow, Poland. The level of RLN identification without the use of IONM in 2011–2014 was assessed in total and near-total thyroidectomies and in totally resected lobes in Dunhill’s operation. RLN identification in subtotal thyroidectomies was excluded from these assessments, because in most of these cases, the RLN was not routinely identified.

A typical cervicotomy was performed in primary thyroid’s operations; in secondary operations, a standard cervicotomy with excision of the existing scar was performed. Usually, an anterior approach between the strap muscles was used for primary thyroidectomy. In reoperations, the lateral approach (between the strap muscles and the sternocleidomastoid muscle) was routinely used. In operations without IONM, the first step was visual identification of the RLN low in the neck (below the crossing with the inferior thyroid artery, we used the inferior thyroid artery as a landmark in visual identification). Once the nerve was visually identified, it was carefully dissected along its course towards the larynx. In operations with IONM, the visual identification of the RLN was facilitated via the IONM system, with the nerve mapping technique. Once the nerve was visually identified, repeated stimulations with the monopolar probe of the IONM system served to trace the nerve path in the operative field and test its functional integrity during dissection. In each patient, the RLN was exposed and the branches of the superior and inferior thyroid arteries were divided close to the thyroid capsule (peripheral ligation).

RLN monitoring was carried out according to the recommendations of the International Neural Monitoring Study Group [5] employing a NIM-3.0 nerve monitor (Medtronic, Jacksonville, USA) and an intermittent IONM technique. A monopolar stimulating probe was used for nerve stimulation with a current amplitude of 1 mA (range 0.5–1.5 mA) and 3-Hz impulses of 200 ms each for 1–2 s. Both the demographic data and the surgical documentation of IONM use were collected in a computerized medical database.

The postoperative follow-up was closely monitored in all the patients. Functional assessment of the larynx was performed on the first postoperative day by an ENT specialist using indirect laryngoscopy. The mobility of the vocal cords in patients with postoperative dysfunction was evaluated by videolaryngoscopy performed up to 6 months postoperatively. Cases in which the function of the vocal cords was recovered within 6 months were considered transient paresis, while an absence of vocal cord mobility 6 months after the operation was considered permanent palsy.

Prism 5.0 statistical software (GraphPad, La Jolla, CA, US) was utilized to analyze the data. Relationships between clinicopathological parameters were analyzed by Fisher’s exact test and the *χ*2 test. To compare the parametrical data, the unpaired Student’s *t* test was used, while the Mann-Whitney *U* test was used to compare groups of data which did not met the assumptions of the parametric test. To compare more than two groups, the Kruskal-Wallis test with post hoc analysis using Dunn’s Multiple Comparison was utilized. In all the analyses, the results were considered statistically significant when *p* < 0.05.

## Results

### Primary endpoint analysis: RLN identification in total or near-total resection of thyroid lobes

Among 236 patients undergoing procedures with IONM (434 RLNs at risk), the RLN was positively identified in 94.93 %.

In 2012–2014, after three 3-month periods of exposition to neuromonitoring, there was an increase in the surgeons’ ability to visually identify the RLN in total resection of thyroid lobes without IONM (*p* < 0.0001). The largest increase in RLN identification skills was observed after the first 3 months of working with IONM in 2012 (101 thyroid operations; *p* < 0.0001). Increases in RLN-identifying skills were also observed after the second (*n* = 70) and third (*n* = 65) periods of IONM utilization, but the values were not at the level of statistical significance (*p* = 0.2929 and *p* = 1.0000, respectively). The 3-year increase achieved statistical significance (*p* < 0.0001) in the surgeons’ ability to visually identify the RLN was observed in primary thyroid operations, while among reoperations, the increase was not at the level of statistical significance (*p* = 0.4268) (Table 2).

**Table 2:** Visual RLN identification before and after exposure to training with intraoperative neuromonitoring

Period and experience with IONM	The year before introduction of IONM	After 1st exposure to IONM	After 2nd exposure to IONM	After 3rd exposure to IONM
Operations with IONM, no. (RLNs at risk) [period]	None [01-12/2011]	101 (190) [01-03/2012]	70 (124) [01-03/2013]	65 (120) [01-03/2014]
Operations without IONM, no. (RLNs at risk in) [period]	119 (235) [01-12/2011]	93 (166) [04-12/2012]	108 (188) [04-12/2013]	76 (138) [04-12/2014]
RLNs at risk in total, near-total thyroid surgery without IONM (%) [period]	140 (100) [01-12/2011]	150 (100) [04-12/2012]	185 (100) [04-12/2013]	138 (100) [04-12/2014]
Visual RLN identification in total, near-total thyroid surgery, no. (%)	64 (45.71 %)	130 (86.66 %)	168 (90.81 %)	126 (91.3)
	*p* < 0.0001^a^ [2011 vs. 2012–2014]			
	*p* < 0.0001^b^ [2011 vs. 2012]			
	*p* = 0.2929^b^ [2012 vs. 2013]			
	*p* = 1.000^b^ [2013 vs. 2014]			
Primary thyroid operations without IONM, no. (RLN at risk)	108 (213)	86 (153)	104 (181)	74 (135)
RLNs at risk in total, near-total primary thyroid surgery without IONM (%)	121 (100)	138 (100)	178 (100)	135 (100)
RLN visual identification in total, near-total primary thyroid surgery, no. (%)	56 (46.8)	121 (87.68)	166 (93.25)	125 (92.59)
	*p* < 0.0001^a^ [2011 vs. 2012–2014]			
Thyroid reoperations without IONM, no. (RLNs at risk)	11 (22)	7 (13)	4 (7)	2 (3)
RLN visual identification in total, near-total thyroid reoperations, no. (%)	8 (42.1)	9 (75)	2 (28.57)	1 (33.33)
	*p* = 0.4268^a^ [2011 vs. 2012–2014]			

*p* value <0.05 was considered statistically significant

*IONM* intraoperative neuromonitoring, *RLN* recurrent laryngeal nerve

^a^Chi-square test

^b^Fisher’s exact test

In patients with non-malignant thyroid disorders that were operated radically, the visual RLN identification rate in 2011, before the introduction of IONM, was 49.21 %; after the experience of working with IONM, the rate of visual RLN identification in this group of patients was 89.21 % in 2012, 97.53 % in 2013, and 91.94 % in 2014. This increase was statistically significant (*p* < 0.0001). A lower percentage of correct visual RLN identification was observed in patients with thyroid cancer: 14.29 % in 2011, 66.67 % in 2012, 62.5 % in 2013, and 85.71 % in 2014; however, even in this group of patients, the increase was statistically significant over the period 2011–2014 (*p* = 0.001).

### Secondary endpoints: the rate of RLN injury and utilization of total thyroidectomy

In 2011–2014, the total rate of RLN palsy in the immediate postoperative period of procedures without IONM fell to a statistically significant degree after three 3-month exposures to neuromonitoring (*p* = 0.043). After each subsequent exposure to IONM, the incidence of paralysis decreased, but the decreases observed from year to year were not at the level of statistical significance (*p* > 0.05) (Table 3). Decreases were also observed in both transient and permanent injury in the years 2011–2014, as well as between successive pairs of years after exposure to IONM, but these decreases were not statistically significant (*p* > 0.05). A steady downward trend in the incidence of both transient and permanent injury was clearly visible in the period 2011–2014. Bilateral paresis occurred in two patients (1.68 %) in 2011 before the introduction of IONM, in one patient (1.07 %) in 2012, and in one (0.92 %) in 2013; all of these were cases of transient paresis.

**Table 3:** Prevalence of RLN injury

Period and experience with IONM	The year before introduction of IONM	After 1st exposure to IONM	After 2nd exposure to IONM	After 3rd exposure to IONM
Operations with IONM, no. (RLNs at risk) [period]	None [01-12/2011]	101 (190) [01-03/2012]	70 (124) [01-03/2013]	65 (120) [01-03/2014]
Operations without IONM, no. (RLNs at risk) [period]	119 (235) [01-12/2011]	93 (166) [04-12/2012]	108 (188) [04-12/2013]	76 (138) [04-12/2014]
Overall RLN paresis, no. (%)	16 (6.80)	6 (3.61)	5 (2.65)	2 (1.45)
	*p* = 0.043^a^ [2011 vs. 2011–2014]			
	*p* = 0.188^b^ [2011 vs. 2012]			
	*p* = 0.761^b^ [2012 vs. 2013]			
	*p* = 0.703^b^ [2013 vs. 2014]			
Transient RLN paresis, no. (%)	10 (4.25)	4 (2.41)	3 (1.59)	2 (1.45)
	*p* = 0.260^a^ [2011 vs. 2012-2014]			
	*p* = 0.321^b^ [2011 vs. 2012]			
	*p* = 0.583^b^ [2012 vs. 2013]			
	*p* = 0.915^b^ [2013 vs. 2014]			
Permanent RLN palsy, no. (%)	6 (2.55)	2 (1.20)	2 (1.06)	0 (0.00)
	*p* = 0.212^a^ [2011 vs. 2012-2014]			
	*p* = 0.342^b^ [2011 vs. 2012]			
	*p* = 0.900^b^ [2012 vs. 2013]			
	*p* = 0.224^b^ [2013 vs. 2014]			

*p* value <0.05 was considered statistically significant

*IONM* intraoperative neuromonitoring, *RLN* recurrent laryngeal nerve

^a^Chi-square test

^b^Fisher’s exact test

Over the 4 years, after three exposures to IONM, the type of thyroid surgery procedures performed without IONM changed. The number of total thyroidectomies rose, while the number of partial procedures declined (*p* < 0.0001). The biggest change took place between 2011 and 2012, after the first 101 thyroid operations performed with IONM (*p* < 0.0001). The same trend continued following the second 3-month period working with IONM (*p* = 0.0019). No statistically significant change was observed in the type of procedure between 2013 and 2014, after the third exposure to IONM (*p* > 0.05); in 2014, partial procedures had been completely replaced by radical operations (Table 4).

**Table 4:** Surgical procedures before and after training with intraoperative neuromonitoring

Period and experience with IONM	The year before introduction of IONM	After 1st exposure to IONM	After 2nd exposure to IONM	After 3rd exposure to IONM
Operations with IONM, no. (RLNs at risk) [period]	None [01-12/2011]	101 (190) [01-03/2012]	70 (124) [01-03/2013]	65 (120) [01-03/2014]
Operations without IONM, no. (RLNs at risk) [period]	119 (235) [01-12/2011]	93 (166) [04-12/2012]	108 (188) [04-12/2013]	76 (138) [04-12/2014]
Partial resection, no. (%)	62 (52.1)	13 (13.98)	2 (1.85)	0 (0.00 %)
- Subtotal thyroidectomy	33 (27.73)	5 (5.38)	1 (0.92)	0 (0.00)
- Dunhill operation	29 (24.37)	8 (8.6)	1 (0.92)	0 (0.00)
Total resection	57 (47.9)	80 (86.02)	106 (98.15)	76 (100)
- Total thyroidectomy	34 (28.57)	53 (56.99)	70 (64.81)	56 (73.69)
- Near-total thyroidectomy	20 (16.81)	8 (8.6)	8 (7.41)	6 (7.89)
- Lobectomy	3 (2.52)	19 (20.43)	28 (25.93)	14 (18.42)
*p* value	*p* < 0.0001^a^ [2011 vs. 2012-2014]			
	*p* < 0.0001^b^ [2011 vs. 2012]			
	*p* = 0.0019^b^ [2012 vs. 2013]			
	*p* = 0.5125^b^ [2013 vs. 2014] ns			

*p* value <0.05 was considered statistically significant. All values represent numbers (%) unless otherwise indicated

*IONM* intraoperative neuromonitoring, *ns* non-significant

^a^Chi-square test

^b^Fisher’s exact test

The mean operative time for thyroidectomies using visual RLN identification decreased following the introduction of IONM and subsequent periods of exposure to neuromonitoring; it was 121 min (±20.6) in 2012, 108 min (±34.08) in 2013, and 94 min (±28.56) in 2014 (*p* < 0.001).

## Discussion

In 1938, Lahey stated that careful dissection of the RLN does not increase the rate of RLN injury during thyroid surgery but definitely reduces the frequency of such injuries; that was the beginning of a new era in thyroid surgery [12]. The need for RLN identification was also confirmed by a 1994 multicenter study by Jatzko et al., which, on the basis of 12,211 thyroid operations, demonstrated that in patients without visual RLN identification, the rate of transient and permanent paralysis was 7.9 and 5.2 %, respectively; this was significantly higher than in the group with visualization of the nerve, where the rates were, respectively, 2.7 and 1.2 %. [4]. Currently, RLN identification is the gold standard in thyroid surgery [5, 6, 13]; we no longer wonder whether to identify the RLN during thyroid surgery, but how to learn to do it successfully. Up until now, more experienced surgeons have taught colleagues how to identify the RLN [14], or magnifying glasses have been used to facilitate its identification. However, it is not always easy; Sturnilo et al. pointed out that in approximately 18 % of patients, localization of the nerve during thyroid surgery failed on both sides; the failure rate climbed to as much as 42 % in reoperations [15].

Given the difficulty of identifying the RLN, the introduction of intraoperative neuromonitoring in 1966 by Shedd [16] seems to be the next breakthrough in thyroid surgery. A number of reports have been published indicating that with IONM, RLN identification reaches 98–100 % [13, 17–20]. Furthermore, several publications have reported a reduction in postoperative complications when utilizing IONM. Barczyński et al. showed that when IONM was used, the incidence of early paralysis was 2.9 % lower in high-risk operations and 0.9 % lower in low-risk surgery compared to the same procedures performed without IONM [13]. Therefore, the optimum would be to use IONM in all thyroid surgery, but currently, only some facilities can afford it for economic reasons [21–23]. In Poland, in 2011, the first conference of the Polish Study Group for IONM of the Polish Club of Endocrine Surgeons took place, and its members agreed upon the need for selective use of neuromonitoring in at least challenging thyroid operations and for education of residents in training [24]. Since then, a gradual but steady increase in the use of this technique has been observed in Poland. Given the financial constraints limiting the widespread use of IONM in thyroid surgery, it may be worth considering ensuring surgeons at least brief exposure to IONM.

The aim of this study was to evaluate the effects of working with IONM as part of a surgeon’s training in the skill of RLN identification, as well as evaluating the quality of surgical treatment after the surgeons’ exposure to IONM. For this purpose, the authors used IONM for 3-month periods for three consecutive years, and the skills gained while working with IONM were then used in operations without IONM during the study period.

Although the surgeons participating in the study carry out about 60 thyroid operations annually, they identified the RLN in only 45.71 % of the cases in the period before the introduction of IONM. This resulted from the surgeons’ inexperience in RLN identification due to the fairly high percentage of partial thyroid procedures performed prior to the introduction of neuromonitoring (52.1 %). In partial procedures, the nerve was not routinely identified; while in complete resections, the technique of carefully following the capsule of the thyroid gland was used.

It turned out that after the first 3-month exposure to IONM—i.e., after 101 procedures—the rate of visual RLN identification rose from 45.71 to 86.66 % (*p* < 0.0001). Further experience with IONM in 2013 and 2014 resulted in additional increases in the identification rate, to 90.81 and 91.3 %, respectively, but these increases were not at the level of statistical significance. Considering the whole 3-year period of working with IONM, the increase in RLN identification was statistically significant (*p* < 0.0001). This shows that IONM is a good tool for learning RLN identification and that it takes only 100 monitored procedures to achieve this goal. But it should be noted that increase in the surgeons’ visual RLN identification abilities was noted only in primary operations (*p* < 0.0001) and not in reoperations (*p* = 0.4268). This fact can be interpreted in two ways. Perhaps, the number of operations with IONM was insufficient to learn accurate visual identification in cases of recurrent goiter; on the other hand, perhaps, reoperations require unconditional use of IONM due to the varying position of the RLN in such cases. This aspect requires further study.

The use of IONM—in particular mapping the RLN and the possibility of electrophysiological confirmation that we have found the RLN—undoubtedly contributed to the development of the authors’ skills in visual RLN identification in operations without neuromonitoring. Using IONM allowed us to learn not only about the typical course of the nerve but also its anatomical variants as positions relative to the inferior thyroid artery or branching. Mapping the RLN and electrophysiological confirmation was especially useful in reoperations, in which the nerve could be dislocated or imbedded in a scar.

It is difficult to relate the results obtained in the present study to other papers on a similar subject, because so far, most of them have focused solely on assessing the IONM method [13, 21], not on its educational value. However, an interesting study by Duclos et al. is worth noting; in that study, it was found that even surgeons with extensive experience in thyroid surgery benefited from the change in their surgical technique after exposure to IONM [25].

Identification of the RLN is reflected both in the rate of injuries to it and the extent of the surgery performed, especially in benign multinodular goiter.

The authors of this study, prior to the introduction of IONM, reported a fairly high percentage of early complications: 6.8 %, including 4.25 % transient and 2.55 % permanent. The first 3-month exposure to IONM resulted in a considerable decline in early RLN paralysis in subsequent operations without neuromonitoring (3.61 %), although the reduction in these complications was not statistically significant. Interestingly, the downward trend in the incidence of RLN paralysis in non-monitored procedures persisted after further exposure to IONM in 2013 and 2014, when the rate of early paralysis dropped to 2.65 and 1.45 %, respectively. However, it was only after 3 years of IONM use that the observed decrease in the incidence of RLN injuries reached the level of statistical significance (*p* = 0.043). This shows that as RLN identification skills increase, the rate of postoperative nerve injury decreased. It needs to be noted, however, that while the exposure to IONM fairly quickly resulted in a statistically significant increase in the surgeons’ ability to identify the RLN—after only 101 operations—it took 3 years and more than 200 thyroid procedures to attain a statistically significant reduction in RLN paralysis.

The authors also assessed the change in the scope of the surgical procedures performed with RLN visualization, but after the experience of working with neuromonitoring. In recent years, total thyroidectomy is increasingly reported as the preferred treatment in nodular goiter [26, 27], but an inability to identify the RLN can lead to a conscious choice of partial resection procedures. This was the case for the authors of this study. In 2011, before the introduction of IONM, more than half of the operations carried out in the authors’ department were bilateral subtotal thyroidectomy or Dunhill’s procedure. After each subsequent year of working with IONM, and as the surgeons’ ability to identify the RLN increased, the extent of thyroid operations also increased. The change in the type of procedures performed was the largest after the first 2 years of experience with IONM, when total resections accounted for 86.02 % (2012) and 98.15 % (2013) of the procedures carried out. These increases were statistically significant (*p* = 0.0019). The third exposure to IONM did not result in any further significant change in the types of procedure; partial removal of the thyroid gland was by then almost completely eliminated. The greater extent of the thyroid resections was not associated with higher rates of RLN injuries.

The results obtained in the present study are very similar to those presented by Dralle et al. in an article form 2014, which is a summary of the results of thyroid surgery in Germany between 2005 and 2011 [28]. This large multicenter study showed that over the years, the percentage of total resection procedures in relation to partial resections rose to 59 % in men and 64 % in women. Also, it was found that with increase in the number of total resections, there was a decrease in postoperative RLN injury, from 1.06 to 0.86 %. It is worth noting that in Germany, most thyroid procedures have been performed with IONM for over 10 years. That multicenter study indicates the directions thyroid surgery takes after the introduction IONM, and the data from the present study confirm that trend: greater extent of surgery with fewer complications.

A study by Alesina et al. about the educational value of IONM is worth mentioning. In that paper, the RLN injury rate during thyroid surgery performed by an inexperienced surgeons without experienced oversight using IONM was 2.7 %, and the results were no worse than those obtained during surgery performed by a an inexperienced surgeons assisted by an experienced surgeon: 2.6 % [29]. Of course, that work does not indicate that IONM can replace experienced assistance during thyroid surgery, but it certainly illustrates the great value of IONM in the process of learning to identify and preserve the RLN.

The results of this study indicate how important even brief exposure to IONM can be. It appears that the greatest advances in RLN identification and the ability to completely remove the thyroid occur after the first, relatively short exposure to IONM—after only about 100 procedures. Statistically significant reductions in the prevalence of RLN injuries come later; it takes more than 200 operations with IONM to achieve that.

Finally, it is worth mentioning that the authors are aware that this study has some limitations. Both the very low rate of visual RLN identification at our center before 2012 and the training in Krakow could have influenced the final results. The very low rate of visual RLN identification at our center before 2012 was due to the large number of subtotal thyroid operations, where the recurrent laryngeal nerve was not identified in most cases. It is the reason why the surgeons have a small experience in visual RLN identification. An additional limitation of this type of study is that the rate of visual identification of the RLN is purely subjective, based solely on the surgeon saying that they saw the nerve in typical localisation using standard approaches to the nerve and typical landmarks to find the nerve. That is a major potential confounding factor in the study.

Despite these limitations, the results show the crucial influence of neuromonitoring on the surgeons’ skills and on the change in surgical thyroid strategy.

In addition, it would be valuable to compare the results among patients operated with and without IONM, but after initial training with a focus on such aspects as the rate of RLN identification, the number of complications, and the duration of thyroid operations, but the issue is a separate ongoing study.

## Conclusions

Three months of exposure to IONM each year from 2012 to 2014 allowed for an increase in RLN identification in totally or near totally resected lobes of the thyroid (*p* < 0.0001), a decrease in the prevalence of RLN injury (*p* < 0.05), and an increase in the safe utilization of total thyroidectomy (*p* < 0.0001) in non-monitored thyroid operations. Thus, IONM is a valuable tool for surgical training.
